# Homozygous nonsense and frameshift mutations of the ACTH receptor in children with familial glucocorticoid deficiency (FGD) are not associated with long-term mineralocorticoid deficiency

**DOI:** 10.1111/j.1365-2265.2008.03511.x

**Published:** 2009-08

**Authors:** Li F Chan, Louise A Metherell, Heiko Krude, Colin Ball, Stephen M P O'Riordan, Colm Costigan, Sally A Lynch, Martin O Savage, Paolo Cavarzere, Adrian J L Clark

**Affiliations:** * Centre for Endocrinology, William Harvey Research Institute, St. Bartholomew's and The Royal London School of Medicine and DentistryLondon EC1M 6BQ, UK; † Institute of Experimental Paediatric Endocrinology, Charite-University medicineBerlin, Germany; ‡ Department of Child Health, King's College HospitalDenmark Hill, London SE5 9RS, UK; § Institute of Child Health, University College LondonLondon WC1N 1EH, UK; ¶ Our Lady's Children's Hospital and The National Centre for Medical GeneticsCrumlin, Dublin, Ireland; ** Department of Paediatrics, University of VeronaVerona, Italy

## Abstract

**Objective:**

Familial glucocorticoid deficiency (FGD) is a rare autosomal recessive disease characterized by isolated glucocorticoid deficiency with preserved mineralocorticoid secretion. Mutations in the ACTH receptor (MC2R) account for approximately 25% of all FGD cases, but since these are usually missense mutations, a degree of receptor function is frequently retained. A recent report, however, suggested that disturbances in the renin–aldosterone axis were seen in some patients with potentially more severe MC2R mutations. Furthermore, MC2R knock out mice have overt aldosterone deficiency and hyperkalaemia despite preservation of a normal zona glomerulosa. We wished to determine whether a group of patients with severe nonsense mutations of the MC2R exhibited evidence of mineralocorticoid deficiency, thereby challenging the conventional diagnostic feature of FGD which might result in diagnostic misclassification.

**Design:**

Clinical review of patients with nonsense MC2R mutations.

**Patients:**

Between 1993 and 2008, 164 patients with FGD were screened for mutations in the MC2R. Totally 42 patients (34 families) were found to have mutations in the MC2R. Of these, 6 patients (4 families) were found to have homozygous nonsense or frameshift mutations.

**Results:**

Mild disturbances in the renin–angiotensin–aldosterone axis were noted in four out of six patients, ranging from slightly elevated plasma renin levels to low aldosterone levels, although frank mineralocorticoid deficiency or electrolyte disturbance were not found. No patient required fludrocortisone replacement.

**Conclusion:**

Severe nonsense and frameshift MC2R mutations are not associated with clinically significant mineralocorticoid deficiency and are thus unlikely to require long-term mineralocorticoid replacement.

## Introduction

Familial glucocorticoid deficiency (FGD) (OMIM: #202200), otherwise known as hereditary unresponsiveness to ACTH, is a rare autosomal recessive disease characterized by isolated glucocorticoid deficiency in the absence of mineralocorticoid deficiency. Patients usually present in early childhood with symptoms associated with cortisol deficiency including recurrent illnesses/infections, hypoglycaemia, convulsions, failure to thrive and shock.^1^ Biochemical assessment will typically reveal extremely high plasma ACTH levels with associated low to undetectable serum cortisol and normal renin and aldosterone levels.^1^–^5^

Inactivating mutations in the ACTH receptor/MC2R in patients with FGD were first described in 1993.^2^,^3^ Since then many other mutations have been identified which account for approximately 25% of cases of FGD (reviewed in Ref. [1]). These mutations are scattered throughout the receptor and the majority are homozygous or compound heterozygous missense mutations leading to substitution of a single amino acid by another. *In vitro*, missense mutations have been shown to result in varying degrees of impaired ACTH signalling, most often by affecting trafficking of the receptor to the cell surface, but also by disruption of ligand binding and loss of signal transduction.^4^–^7^ In comparison, homozygous nonsense and frameshift mutations are extremely rare and only one such mutation has been reported.^8^ The expected result of such mutations would be a significantly truncated protein severely disrupting MC2R function.^9^ Assessment of one truncating mutation of this type, the 652insA mutation (insertion of a single adenine resulting in a frameshift after the glycine residue at amino acid position 217), revealed severely impaired function of this mutant receptor.^6^ This raises the possibility that nonsense or frameshift mutations of the MC2R may be associated with a distinct clinical phenotype with the consequence that these patients are not classified as having FGD on clinical criteria.

The classical phenotype of FGD is that of isolated glucocorticoid deficiency. However, it has long been recognized that minor impairment of the renin–angiotensin–aldosterone axis is seen in some patients at the time of presentation.^10^,^11^ The significance of this observation is unclear as the changes are usually transient and mineralocorticoid treatment is not required. In normal human physiology, aldosterone secretion is primarily determined by the levels of angiotensin II. However, ACTH acting through the MC2R in the zona glomerulosa (ZG) is an efficient stimulus to aldosterone secretion, and administration of synthetic ACTH (1-24) to normal human subjects results in a rise in plasma aldosterone levels.^11^ Failure of this path – especially in times of stress – may impair aldosterone production.

Recently published data on the MC2R knockout (KO) mice revealed that these mice have low serum aldosterone levels leading to hyperkalaemia in females, and increased expression of the angiotensin receptor 1b (AT1bR) in the ZG, implying an element of compensation in the renin–angiotensin–aldosterone axis in the complete absence of MC2R.^12^,^13^ Furthermore, Lin *et al*. recently described a series of children with MC2R mutations who had a degree of increased renin activity or aldosterone deficiency at initial investigation and were treated with 9α-fludrocortisone. Amongst these was one child with a homozygous deletion in the MC2R leading to a nonfunctional receptor and who had elevated plasma renin activity (PRA), low aldosterone concentrations and electrolyte disturbance.^8^

These data suggest that human MC2R mutations which result in the total absence of functional MC2R may result in disruption of renin–angiotensin–aldosterone axis and potentially the categorization of these patients as having adrenal hypoplasia rather than FGD. In an attempt to examine this hypothesis we identified six previously unreported patients (out of 164 FGD patients) with novel frameshift and nonsense MC2R mutations, and retrospectively sought evidence of mineralocorticoid deficiency.

## Methods

### Patients

A total of 164 FGD patients referred from national and international centres from 1993 to 2008 were screened in our laboratory for mutations in MC2R. All patients had elevated plasma ACTH and low to undetectable serum cortisol levels. Forty-two individuals from 34 families were found to have mutations in the MC2R (25·6%). Half of these were homozygous S74I missense mutations (21 patients from 16 families).^2^ Among the group of patients screened, six patients from four families were identified with homozygous nonsense or frameshift mutations. Clinical details and their endocrine investigations are summarized in Table 1. In all cases, informed consent for mutational analysis was obtained. The study was approved by the local institutional ethic committee.

**Table 1:** Clinical and biochemical data on all six patients with homozygous MC2R nonsense and frameshift mutations

Family		I	II		III	IV	
Patient number		1	1	2	1	1	2
MC2R mutation		c.459–460insC	c.634delA	c.634delA	c.702delC	c.539C >	c.539C > A
		(p.I154fsX248)	(p.R212fsX215)	(p.R212fsX215)	(p.F235fsX241)	A (p.S180X)	(p.S180X)
Sex		M	F	M	M	M	F
Age of first S/D		3 days/7 months	10 days/1 month	16 months	7 weeks	2 days/4 weeks	6 days
Age at Last F		10·8 years	4·8 years	2·7 years	1 year	15 years	12 years
Presentation		Neonatal hepatitis, hyperpigmentation, prolonged jaundice	Hypoglycaemic seizures, sepsis	Hypoglycaemic seizures	Hypoglycaemia, hyperpigmentation, prolonged jaundice, FTT	Hypoglycaemic seizures, hyperpigmentation	Hypoglycaemic seizures Hyperpigmentation,
Cortisol (nmol/l)	D	< 30	< 30	< 30	< 30	< 30	< 30
(N.R. 120–620)							
ACTH (pg/ml)	D	639	–	> 1250	> 1250	> 3000	> 3000
(N.R. < 50)	F	7790	< 5†	< 6†	NA	NA	–
Aldosterone (pmol/l)	D	100‡	470	320	NA	NA	939
	F	**< 70**	190	650	**70/290**	NA	103
N.R.		100–450	100–440	83–840	300–2200	NA	83–941
Plasma renin/plasma	D	4·5 pmol/ml/h‡	**433 µU/ml**	**63 µU/ml**	NA	NA	**> 19 pmol/ml/h**
renin activity	F	**6·7 pmol/ml/h**	**68·7 µU/ml**	**80·2 µU/ml**	3·5 pmol/ml/h, 5·6 pmol/ml/h	**0·6–9·1 pmol/ml/h**	12·8 ng/l (7·4–56·2)§
N.R.		2·8–4·5	3·9–49·3	3·3–41	1·3–8·6	1·2–5·3	1·2–5·3
UE at D and F		Na +132 at D, Normal at F	Normal	Normal	Normal	Normal	Normal
Treatment (mg/m^2^/day)		HC 10·2	HC 19·5	HC 23·4	HC 20	HC 12·9	HC 14·5

SI conversion: Aldosterone, pmol/l = 27·7 ng/dL, PRA, pmol/h/ml = 0·77 ng/h/ml, was used where appropriate. D, at diagnosis; HC, hydrocortisone; UE, urea and electrolytes; S, symptoms; FTT, failure to thrive; F, follow-up; NR, normal range; NA, results not available.

† Suppression of ACTH on high doses of HC.

‡ Results taken age 5·9 years (date of FGD diagnosis). Abnormal renin/aldosterone results in bold.

§ Corresponding NR in ng/l.

### Biochemical investigations

Biochemical investigations were performed at the referring institute; all cases were assessed by a paediatric endocrinologist at a tertiary centre. Age appropriate, centre specific normal values were provided by the lead clinicians and are shown in Table 1.

### Mutational analysis of MC2R

Genomic DNA was extracted from blood leucocytes. PCR of the single coding exon was performed using specific intronic primers (primer sequences and PCR conditions available on request). PCR products were visualized on a 1% agarose gel, and sequenced using the ABI Prism Big Dye Sequencing kit and an ABI 3700 automated DNA sequencer (Applied Biosystems) in accordance with the manufacturer's instructions. Mutations were confirmed on two independent PCRs sequenced in both directions and described in accordance to recommended nomenclature.^14^

## Results

### Patient I:1 –*c.*459_460insC resulting in p.I154fsX248

Patient I:1 presented with neonatal hepatitis and hypoglycaemia at day 3 of life. He was the second child of a consanguineous Kuwaiti family. His 8 years old brother was unaffected. He was treated for presumed sepsis and investigated for his neonatal hepatitis. He was found to have a high TSH and low FT_4_ and diagnosed with hypothyroidism. Treatment was initiated with levothyroxine. There was a family history of thyroid disorders, with Hashimoto thyroiditis (father) and a paternal cousin with panhypopituitarism. He was reassessed at the age of 7 months and diagnosed with glucocorticoid deficiency and initiated on hydrocortisone treatment. Biochemical assessment revealed an undetectable serum cortisol level of < 30 nmol/l during a hypoglycaemic episode (blood glucose was 1·9 mmol/l). Plasma ACTH levels were elevated to 639 ng/l. Serum sodium was 132 mmol/l with a normal potassium level of 4·5 mmol/l. A short synacthen test showed no response to 1–24 ACTH, baseline cortisol was 21 nmol/l and peak of 10 nmol/l. Hyperpigmentation was noted and in hindsight parents had commented on his dark skin soon after birth. Adrenal and thyroid antibodies were negative. A diagnosis of FGD was considered at 5·9 years of age when he was reassessed at a separate centre. While on hydrocortisone replacement, plasma renin levels were deemed ‘very high’ during an admission for a glucagon stimulation test. This was considered to be due to an element of hypovolaemia during the test. Aldosterone levels were normal at 100 pmol/l (N.R. 100–450 pmol/l). Subsequent repeat renin levels performed while clinically well on treatment showed a PRA on the upper range of normal (4·5 pmol/ml/h; N.R. 2·8–4·5 pmol/ml/h). Further assessment at 10·8 years, while on hydrocortisone treatment the patient showed a persistently elevated plasma ACTH level of 7790 ng/l (N.R. < 50 ng/l), typical of FGD. Aldosterone level was low < 70 pmol/l, paired with a slightly high PRA of 6·7 pmol/ml/h, suggestive of a degree of mineralocorticoid deficiency. He remains well on hydrocortisone treatment 10·2 mg/m^2^/day. Mutational analysis of the MC2R revealed a homozygous insertion of a cytosine (C) at nucleotide positions 459–460. At a protein level this would result in a substitution of the isoleucine residue at amino acid position 154 by a histidine and subsequent frameshift leading to a nonsense sequence and premature termination of the receptor at position 248 (p.I154fsX248). Both parents were assessed and found to be heterozygous for the C nucleotide insertion.

### Patients II:1 and II:2 –*c.*634delA resulting in p.R212fsX215

Patients II:1 and II:2 are siblings, diagnosed with primary adrenal insufficiency at the ages of 1 and 16 months, respectively. Parents were first cousins and patient II:1 presented at 10 days of age with neonatal sepsis and severe hypoglycaemia. Biochemical investigations revealed an undetectable cortisol of < 30 nmol/l (N.R. 120–620 nmol/l). At diagnosis plasma renin concentration was elevated at 433 µU/ml (N.R. 3·9–49·3 µU/ml) while aldosterone was 470 pmol/l (N.R. 100–440 pmol/l). Serum electrolytes were normal. Glucocorticoid replacement therapy was initiated soon afterwards. Reassessment while on treatment showed an elevation of renin levels of 68·7 µU/ml, while aldosterone was normal 190 pmol/l. At 4·8 years of age she developed obstructive-hypertrophic cardiomyopathy necessitating treatment with Verapamil. She continues to grow well, weight > 99th centile and 75th centile for height. Her brother (patient II:2) presented at 16 months of age with hypoglycaemic seizures. Biochemical assays at diagnosis revealed an elevated ACTH level of > 1250 pg/ml (N.R. < 50) and undetectable cortisol. As with his sibling, his renin level was elevated to 63 µU/ml (N.R. 3·3–41 µU/ml), with normal Aldosterone (320 pmol/l; N.R. 83–840). Renin levels remained high despite relatively high doses of hydrocortisone replacement. Plasma renin concentration was 80·20 µU/ml and aldosterone 650 pmol/l. He remains clinically well on treatment with hydrocortisone 23 mg/m^2^/day. Mutation analysis of both affected children revealed a novel homozygous 634delA mutation leading to a frameshift at position 212 and an early stop codon at position 215 (p.R212fsX215). Parents were heterozygous for this variant.

### Patient III:1 –*c.*702delC resulting in p.F235fsX241

Patient III:1 presented at 7 weeks of age with hypoglycaemia, hyperpigmentation, prolonged jaundice and failure to thrive. He is of Sudanese origin and parents are first cousins. Plasma ACTH levels at diagnosis were extremely elevated at > 1250 pg/ml (N.R. < 50 pg/ml) paired with a serum cortisol of < 30 nmol/l. He was hypoglycaemic (blood glucose < 1·1 mmol/l) at diagnosis and a short synacthen test confirmed the diagnosis of adrenal insufficiency, with no cortisol response to 1–24 ACTH. Serum electrolytes were reportedly normal throughout childhood. PRA at age 1·0 years was 3·5 pmol/ml/h (N.R. 1·3–8·6 pmol/ml/h). The corresponding plasma aldosterone levels were low at 70 pmol/l (N.R. 300–2200), and on repeating 1 month later PRA was normal at 5·6 pmol/ml/h while aldosterone remained slightly low at 290 pmol/l (N.R. 300–2200). The child is thriving on treatment with height > 75th centile and weight > 99th centile. Treatment continues at a hydrocortisone dose of 20 mg/m^2^/day. Adrenal autoantibody screen was negative. Mutational analysis of the MC2R revealed a homozygous deletion of a cytosine (C) nucleotide, resulting in a frameshift at amino acid position 235 and premature stop codon.

### Patient IV:1 and IV:2 –*c.*539C>A (p.S180X)

Patients IV:1 and IV:2, siblings of Lebanese origin, both presented with hypoglycaemia and seizures in early childhood. At presentation ACTH levels were high with undetectable cortisol levels. Both children were persistently hyperpigmented with high ACTH levels despite high hydrocortisone replacement dosages. They have had repeated infections associated with seizures requiring numerous episodes in hospital. Patient IV:2 had elevated PRA at diagnosis, > 19 pmol/ml/h (N.R. 1·2–5·3 pmol/ml/h) with a corresponding normal aldosterone level of 939 pmol/l (N.R. 83–941 pmol/l). At follow-up, renin concentration was normal with a corresponding aldosterone level in the low normal ranges (renin at 12·8 ng/l; N.R. 7·4–56·2 ng/l, and plasma aldosterone level 103 pmol/l (N.R. 83–941 pmol/l). Patient IV:1 was noted to have normal to intermittently elevated PRA on treatment (0·6–9·1 pmol/ml/h). Serum electrolytes were normal. On sequencing the MC2R both children have a homozygous nonsense mutation with a nucleotide substitution (C > A) at nucleotide position 539. The result of this is the introduction of a stop codon at position 180 (p.S180X). This would result in a severely truncated protein at this position that lacks the 6th and 7th transmembrane domains, third intracellular loop, third extracellular loop and the C terminal tail.

## Discussion

The diagnosis of FGD is classically based upon the presence of isolated glucocorticoid deficiency and the absence of mineralocorticoid deficiency. Transient changes in sodium homeostasis are known to occur at times of acute illness and can partly explain how patients may be misdiagnosed with salt-losing adrenal hypoplasia.^8^,^10^,^11^ ACTH is generally considered to play a less prominent role compared to angiotensin II in the control of aldosterone production in the human. However the Mc2r^−/–^ mouse suggests a more directly important role. Mc2r^−/–^ mice were shown to have significantly lower, though not undetectable, serum aldosterone levels compared to wild-type litter mates. This is accompanied by increased adrenal AT1bR expression, while CYP11B2 (aldosterone synthase) expression remains low. Renin levels or activity were not measured.^12^,^13^ However these findings contrast with recently published studies on Pomc^−/–^ mice which demonstrate the presence of secondary hyperaldosteronism in the complete absence of ACTH.^15^ The differences between these two models are not entirely clear. The mechanism of hyperaldosteronism in the Pomc^−/–^ model is probably secondary to the hypotensive effect of glucocorticoid deficiency which leads to activation of the renin–angiotensin system. Why this effect is not seen in the Mc2r^−/–^ animals is not clear, but might reflect a developmental role for this receptor in the mouse.

**Fig. 1:**
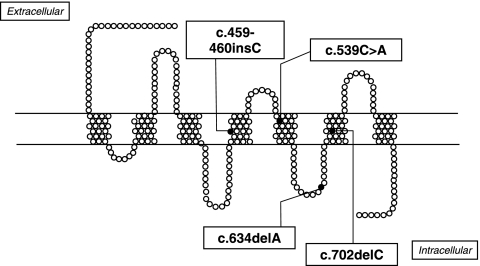
Schematic representation of the MC2R, showing the position of the nonsense and frameshift mutations identified in six patients described in this study (filled circles).

The Mc2r^−/–^ mouse phenotype also appears to contrast with the ‘human equivalent’, that is, FGD patients with MC2R mutations. These patients have normal mineralocorticoid status. One explanation is that the majority of MC2R mutations are missense mutations with some retention of function, and hence do not represent a human equivalent of an MC2R knockout.^1^–^5^,^7^,^8^ Instead, homozygous nonsense/frameshift mutations may represent a more appropriate group to compare with Mc2r^−/–^ mice, although such patients are extremely uncommon. The six patients described in this article represent a unique cohort found to have homozygous frameshift and nonsense mutations of the MC2R (Fig. 1). All six children presented in either the neonatal period or in infancy. Presenting symptoms were typical and include hypoglycaemia, hyperpigmentation, neonatal hepatitis, prolonged jaundice, seizures and failure to thrive. All six had very low or undetectable serum cortisol paired with extremely elevated plasma ACTH levels.

This series demonstrates that some patients with severe MC2R mutations have some degree of disturbance of the renin–angiotensin–aldosterone axis, although no patient was frankly mineralocorticoid deficient or required treatment with 9α-fludrocortisone. Of the six patients described, elevated renin levels or PRA on follow-up were noted in three. The corresponding aldosterone level was low on one occasion on follow-up in one (patient I:1) and slightly low on follow-up in patient III:1 in association with normal renin levels. All patients were well during reassessment. None of the six patients have received treatment with 9α-fludrocortisone. Hydrocortisone has some mineralocorticoid activity and this may be sufficient may blunt evidence of aldosterone deficiency in replaced patients.

Thus while it remains possible that some patients with nonsense mutations may have been diagnosed with either primary adrenal hypoplasia or early onset antibody negative Addison's disease, it seems unlikely that a distinct phenotype resulting from this type of mutation exists.

In conclusion, we believe it is unlikely that a distinct group of patients with inherited failure of adrenal glucocorticoid and mineralocorticoid production results from MC2R defects, and that the classical FGD phenotype with isolated glucocorticoid deficiency is the typical clinical consequence of any inactivating mutation of this receptor.
